# Pathological Discrepancy: Simple Mesenteric Cyst vs. Mesenteric Lymphangioma

**DOI:** 10.1155/2021/8848462

**Published:** 2021-03-18

**Authors:** Vygintas Aliukonis, Marius Lasinskas, Algirdas Pilvelis, Audrius Gradauskas

**Affiliations:** ^1^Department of Abdominal Surgery, Clinic of Surgery, Vilnius City Clinical Hospital, Antakalnio 57, LT-10207 Vilnius, Lithuania; ^2^Centre for Health Ethics, Law and History, Institute of Health Sciences, Faculty of Medicine, Vilnius University, 03101 Vilnius, Lithuania; ^3^Department of Nursing and Fundamentals of Internal Medicine, Faculty of Medicine, Vilnius University, Antakalnio 57, LT-10207 Vilnius, Lithuania

## Abstract

**Introduction:**

Both mesenteric cysts and cystic lymphangiomas are scarce and clinically and radiologically almost identical derivatives, but their histological structure is fundamentally different. *Case Presentation*. A 52-year-old woman was consulted by a surgeon for a derivative felt in her abdomen. The patient said she felt a growing derivative in the abdomen about a month ago. After consulting and testing, a sigmoid colon mesenteric cyst (13 cm × 11 cm × 10 cm) was found. Complete excision of the cyst within healthy tissues was performed through laparotomy. The surgery had no complications. The initial pathological answer was a simple mesothelial cyst (a rare histological finding). However, immunohistochemical tests were performed that showed that diagnosis was mesenteric cystic lymphangioma (ML). Cystic lymphangiomas that have a link to the mesentery have been described less than 200 times.

**Conclusions:**

Final differential diagnosis between different cystic derivatives is possible only based on histopathological examinations. Mesenteric lymphangioma is most common at a very young age, but in rare cases, it also occurs in adults. All clinicians should increase their awareness of the disease.

## 1. Introduction

Peritoneal cysts were first described as early as 1507 by the Italian anatomist Benevenni when a postmortem evaluation was performed. The first successful removal of the peritoneal cyst was completed in 1880 [1].

Both mesenteric cysts and cystic lymphangiomas are very rare and clinically and radiologically almost identical derivatives, but their histological structure is fundamentally different [2].

The main difference demonstrating the need for differential diagnosis lies in the fact that lymphangiomas, although usually benign, have a greater probability of becoming aggressive. They can become invasive and proliferate in the surrounding tissues and organs [3].

## 2. Clinical Case

A 52-year-old woman was consulted by a surgeon for a derivative felt in her abdomen. The patient said she felt a growing derivative in the abdomen about a month ago.

There was also slight abdominal ache and discomfort while sitting and cycling.

**Figure 1:**
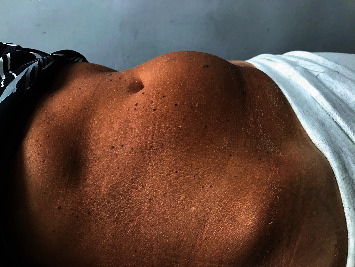
Deformation of the abdominal area.

During the initial examination, deformation of the abdominal wall is observed and a painless derivative is felt in the lower left quadrant of the abdomen (Figure 1). Externally, the object was palpable in the range of about six centimeters. It was stiff and partially mobile.

Ultrasound examination revealed a cystic derivative with clear boundaries.

However, ultrasound is not the first-choice research for assessing the intestine and mesentery derivatives, due to difficulty in assessing a clear relationship with the surrounding tissues and structures.

Therefore, CT scan was performed.

**Figure 2:**
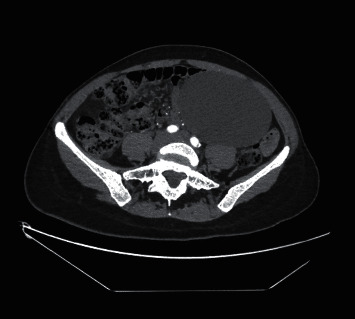
CT scan with a cystic derivative in the left lower abdominal quadrant.

A cystic derivative (13 cm × 11 cm × 10 cm in size) was found in the left lower abdominal quadrant according to the CT data. The derivative has no radiological evidence of malignancy. There were no signs of overgrowth into other structures, as well as no clear contact with large blood vessels. Radiologically, the derivative was most similar to a mesenteric cyst. Mild hydronephrosis due to pressure in the left ureter is also observed (Figure 2).

**Figure 3:**
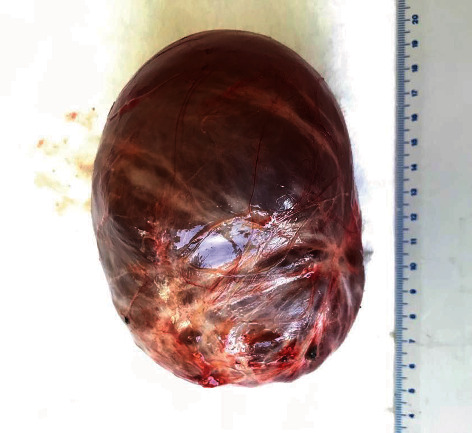
Complete excision of the cyst.

**Figure 4:**
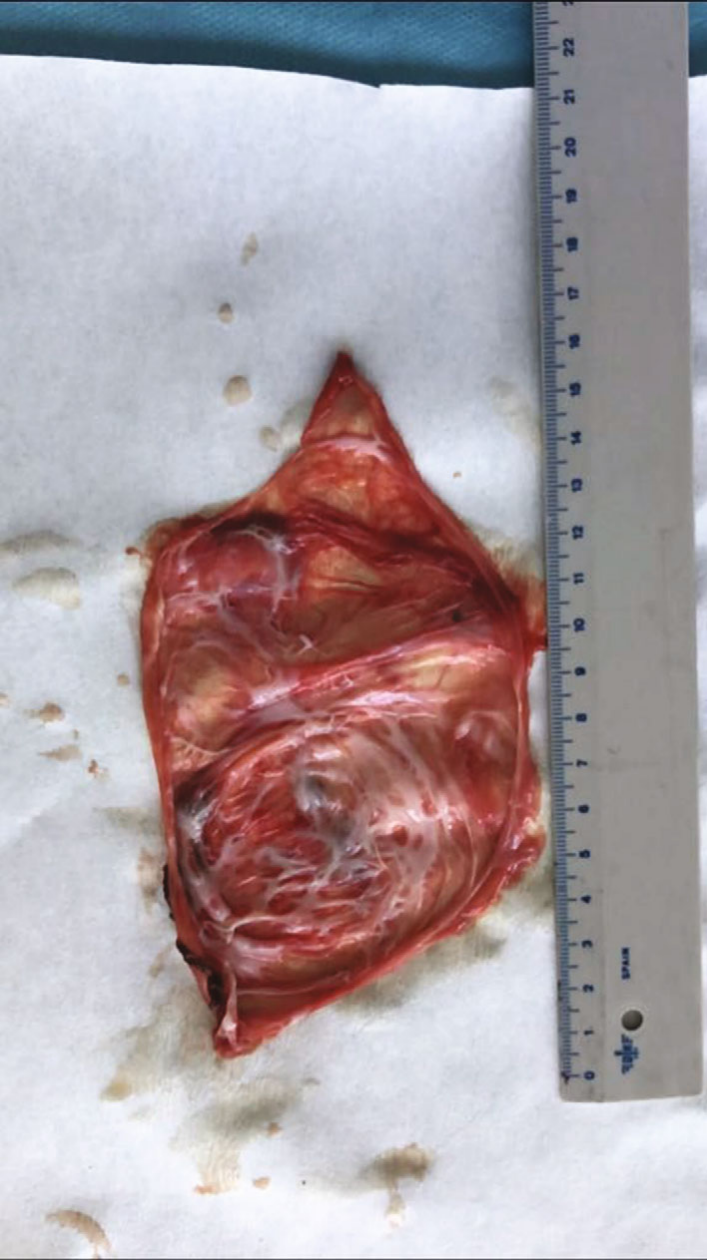
A thin-layered cyst with a focal fusion of the inner monolayer of the epithelium.

The patient underwent laparotomy, during which a movable, thin-walled, cystic derivative (about 12 cm in size) with transparent content in a sigmoid colon mesentery was found. Complete excision of the cyst within healthy tissues was performed, protecting the surrounding blood vessels (Figures 3 and 4).

The surgery had no complications.

The patient was discharged on the fourth postoperative day with an uneventful postoperative process. The patient underwent two ultrasound examinations during the first year of postoperative treatment, one after three months and the other after six months. Both examinations were free of visible pathological changes. One year after the operation, abdominal and pelvic computed tomography was performed; no visible pathology was found.

The pathological findings were a thin-layered cyst with a focal fusion of the inner monolayer of the epithelium.

The conclusion was that this was a simple mesothelial cyst.

**Figure 5:**
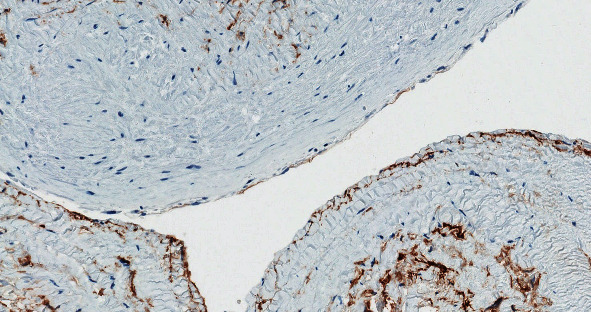
A negative immunohistochemical reaction of CD34 (background-positive internal control-positive staining of small vascular epithelium) precludes the diagnosis of vascular cysts.

**Figure 6:**
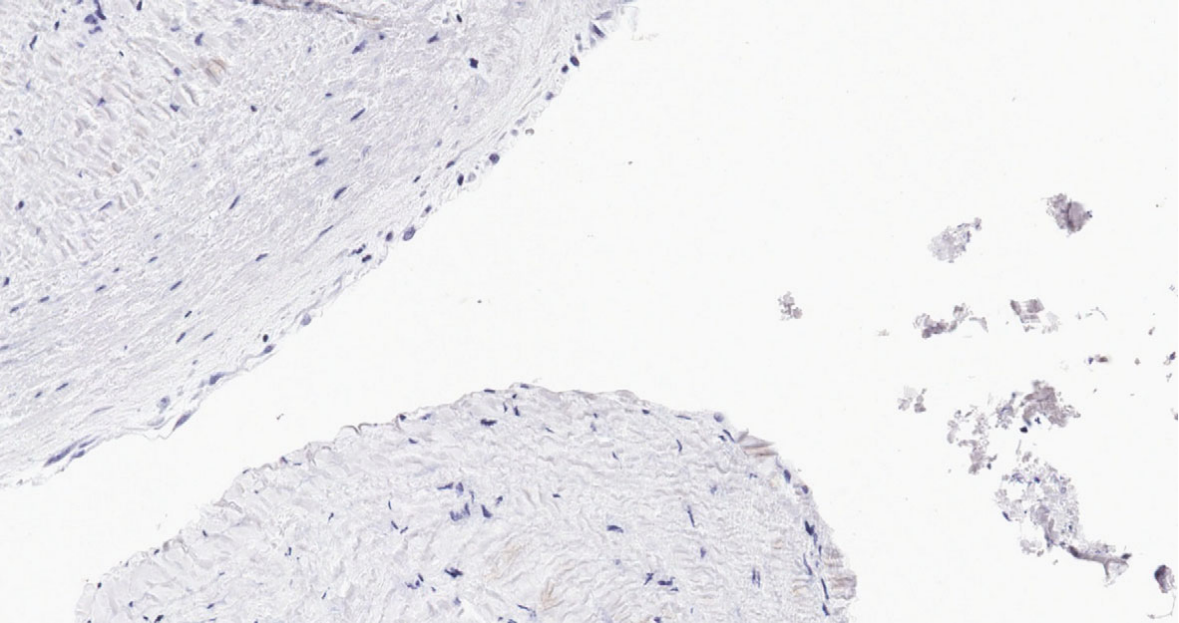
Negative staining of Calretinin rejects the diagnosis of the mesothelial cyst.

However, when the pathologist suspected that the diagnosis might be inaccurate, additional immunohistochemical tests were performed: derivative lining endothelial cells D2-40(+++) (cytoplasmic response), 100%; CD34(+)(cytoplasmic response), 10%; Calretinin(-); WT1(-); CD10(-); panCK(AE1/AE3)(-); CK7(-); and estrogen receptors(-) (Figures 5 and 6).

Immunohistochemical tests showed that the proper diagnosis was mesenteric lymphangioma (ML).

## 3. Discussion

The prevalence of simple mesothelial cyst cases in literature is scarce. The total number of reported cases in the world is less than a thousand. The cases described range in size from a few centimeters to giant cysts reaching 5 liters [4].

The Perrot classification, which is used to classify cystic derivatives, indicates that the simple mesothelial cyst (SMC) is a benign mesenteric mesothelial cyst. Perrot and coauthors proposed to classify cysts according to histopathological features and origin into six groups: lymphatic, mesothelial, intestinal, urogenital, dermoid cysts, and nonpancreatic pseudocystic origins [5].

It is considered that mesothelial cysts are caused by incomplete fusion of mesothelial layers [6].

The common mesothelioma cyst is usually asymptomatic or manifests itself with very nonspecific clinical signs: slight abdominal ache, nausea, etc. The presence or absence of symptoms depends mainly on the location of the cyst in the abdomen. Symptoms are caused by “external pressure” (hydronephrosis, lymphedema, etc.). It is not possible to determine the threshold beyond which the cyst's size already causes symptoms, as this is highly dependent on the localization in which the cyst is located and the anatomical characteristics of the patient. As a result, some people may begin to experience cysts' symptoms while it is just a few inches in size, for example, when it forms near the urethra and pressures it. On the contrary, others may not feel any symptoms until the cyst reaches a large volume if it starts in the “free abdominal area.”

Pain is not a very common symptom, and if it does occur, the first thing to think about is intestinal strangulation [7].

Meanwhile, with regard to cystic lymphangiomas, it should be noted that the disease is so rare that in all of the English literature, cystic lymphangiomas that have a link to the mesentery have been described less than 200 times [8]. It is therefore natural that this type of diagnostic error for two particularly similar and very rare conditions is not uncommon and is described in the literature [9, 10].

Lymphangiomas can occur in any part of the body, most commonly found in the head and neck, and sometimes in the abdominal wall area. The discovery of lymphangiomas in the mesentery is an infrequent event [11]. As a percentage of all lymphangiomas, mesenteric lymphangiomas account for only 11% of all cases [12].

Lymphangiomas are classified into simple, cavernous, and cystic [8].

The etiology of the disease is not known, but it is thought to be related to disorders of lymphatic system formation. Such an embryological theory is supported, at least in part, by the fact that the vast majority of cases are detected as early as childhood [13, 14]. About 90% of all lymphangiomas are found in children under two years of age [15]. Meanwhile, only isolated cases have been reported in adult patients [16].

There are small lymphoid aggregates in the cyst wall that help distinguish between lymphangiomas and simple cysts of the mesentery.

However lymphangiomas might be difficult to distinguish from hemangiomas, and therefore, final diagnosis can only be confirmed immunohistochemically [17].

## 4. Diagnostics

If cystic derivatives in the abdomen are suspected, it is recommended that the first-line examination starts with an ultrasound of the abdominal organs. This examination is inexpensive, harmless, and gives good results for initial assessment (screening) [18]. The only major disadvantage of ultrasound is that it is an examination that is particularly dependent on the experience of a specialist, so it is important to emphasize that ultrasound alone cannot be relied upon.

The disease may mimic the cavernous masses caused by tuberculosis, which is relevant in countries with a high incidence of tuberculosis (for example, Eastern Europe) [19].

Computed tomography or magnetic resonance imaging is recommended for a more detailed evaluation of the derivative before surgery, its interfaces with surrounding organs, and blood vessels [20].

## 5. Treatment

Although isolated studies are showing that active monitoring is an appropriate choice for asymptomatic patients [8], however, most authors agree that first-line treatment should be radical removal of the derivative, even in asymptomatic patients [21].

Undoubtedly, the question may arise about the goal of immediate radical removal of a derivative, which is usually benign, especially considering that spontaneous regression cases of resembling derivatives can also be found in the literature [22]. On the other hand, only a few cases with spontaneous regression of lymphangiomas were described in the literature. While the chances of malignancy are short, but in pathology so rare, no reliable statistics can be made. It should also be emphasized that even in asymptomatic cysts, their localization is exceptionally relevant to the final choice of treatment. Mesenteric cysts, which have much more intense conjunction with the intestines, are also more likely to interfere with function for mechanical reasons (e.g., intestinal volvulus). Finally, even the authors of the publication on self-regression agreed that complete surgical excision is the treatment of choice.

Suggestions are based on the fact that benign derivatives, though exceptionally rare, can change their degree of malignancy. This change might occur not only through the natural course of the disease (mesenteric lymphangioma is at risk of transforming into a sarcoma under the influence of ionizing radiation) but also due to the human factor in histological diagnosis (like in the case we describe) [23].

It should also be noted that there have been described cases where even a simple mesenteric cyst has been reclassified to a malignant cyst with aggressive spread within ten years after six surgical interventions (to reduce the volume) [16].

Laparoscopic methods for the removal of such cysts can be found in the literature [24]. Still, these are isolated, rather selectively selected patients, and the operation itself requires extensive skills in laparoscopic surgery. Open operation is still considered a more acceptable method [25].

In some cases, when surgical treatment is too risky, cyst drainage is described as a method of treatment. However, 1/3 of the patient's cyst recurred quite quickly, not to mention that the cells were not affected in any way, and not all of the complications of malignancy described above were avoided [1, 8, 26].

Drainage on its own is not a cure but rather a bridge to surgical treatment or symptom relief to reduce cyst volume.

Other minimally invasive treatments of lymphangiomas include sclerotherapy with doxycycline or alcohol [27–29].

It must be emphasized that these are isolated cases and not standard clinical practice. To our knowledge, no extensive studies are currently evaluating these methods. Therefore, we cannot predict whether it will be generally used.

## 6. Conclusions

The final differential diagnosis between different cystic derivatives is possible only based on histopathological examinations.

Mesenteric lymphangioma is most common at a very young age, but in rare cases, it also occurs in adults.

Mesenteric lymphangioma is usually nonmalignant but can sometimes lead to very severe consequences.

Timely and radical surgery can completely cure the disease and help prevent malignancy.

Clinicians should increase their awareness of the disease.
